# It Matters Who You Ask: Validity and Reliability of Animal Empathy Scoring Scales in Canadian Public and Participants in Beef Production

**DOI:** 10.3390/ani15121788

**Published:** 2025-06-17

**Authors:** Christy Goldhawk, Ed Pajor

**Affiliations:** Faculty of Veterinary Medicine, University of Calgary, Calgary, AB T2N 1N4, Canada; eapajor@ucalgary.ca

**Keywords:** livestock production, public perspective, calf, handling, welfare

## Abstract

Reliable measurements are central to understanding animal-directed empathy. This research study utilizes data from two online surveys to evaluate the validity and reliability of measures of animal-directed empathy. The survey data was of (1) Canadians who have participated in beef cattle processing events (*n* = 812), and (2) members of the public from across Canada (*n* = 668). As a part of these surveys, individuals were asked 22 animal empathy score (AES) questions, and an additional 5 questions about livestock-directed empathy (LES). The AES correlated well with an 8-question short form (AES-SF) previously developed in other studies. Confirmatory factor analysis showed that the AES-SF structure was a good fit within the public responses but did not fit well with the responses of those participating in the beef industry. The reliability of the AES and AES-SF was high in the public population, but low in the population participating in beef cattle production. The LES fit well with the public responses, with high reliability and moderate correlation with AES; however, it did not fit well within the industry participant responses. Overall, the results support the use of AES-SF as a measure of animal-directed empathy within public populations. Measurement of this construct needs further development for individuals working directly with livestock species. Researchers should proceed with caution in using animal-directed psychometric measures validated with public populations, as evidence from this study suggests these measures have poor reliability and validity in populations of individuals working directly with livestock species.

## Simple Summary

Human empathy towards animals provides insights into how we choose to live, what products we purchase, policies we support, and how we treat animals under our care. The methods used to evaluate our empathy towards animals, however, often focus on general domestic species, are too long to be useful in surveys, or have not been developed for use with those that work with animals. This study found that tools for measuring empathy towards animals were reliable in Canadian public populations, even when shortened. The same tools, however, were unreliable when used to assess empathy towards animals in people that work directly with animals for food production. Thus, when wanting to understand human empathy towards animals it is necessary to be mindful of who is being asked and to use validated and reliable tools for that population.

## 1. Introduction

Empathy towards animals can influence how we choose to live, what products we purchase, policies we support, and how we treat animals under our care [1,2,3,4,5]. The level of empathy can vary by species and biocultural factors of a population [6,7], including how individuals chose to engage with the treatment of non-human animals [8]. Indicators of empathy from individuals involved in livestock production can relate to decisions about animal welfare and how the animals perform in livestock production systems [9]. Assessing attitudes towards animals, inclusive of animal-directed empathy, has been proposed as a valuable proxy measure in benchmarking animal welfare in livestock production due to relationships with human behaviours towards livestock [10,11].

The measurement of indicators of animal-directed empathy often relies on quantitative methods that use self-reporting questions assessing level of agreement with statements related to empathy, such as empathetic concern and perspective taking [12]. These measurements have been developed by re-phrasing questions from human psychometrics to focus on the general subject of “animals” or species-specific subjects such as pets, wildlife, or livestock species. Two common measurements are the 10-item Empathy Towards Animals scale (ETA [13]), which addresses animal-directed empathy through the evaluation of empathetic concern and perspective taking, and the 22-item Animal Empathy Scale (AES), which has a greater emphasis on the emotional component of empathy [14]. The AES has been used in multiple languages and cultures, with high reliability values [15]. The assessment of both measures indicates that these are valid and reliable tools for animal-directed empathy in public populations [15,16].

Multi-item measures of a construct, particularly when used in combination with other survey items, must balance the need to adequately address the participants’ views without causing bias due to fatigue or practical constraints such as cost and lower response rates [4,15]. Due to the length of the AES and interest in shorter forms of psychometrics, ref. [15] used the original AES to extract and validate an eight-item measure of animal empathy (AES-SF). The authors of this study call for testing in other populations to assess the validity and reliability of the shortened measurements.

Many studies use populations of university students or samples from the public in pilot testing questions [13,14,15,17], even when the target population is those working directly with animals in livestock production [18,19]. Studies have also used existing tools to suggest differences in attitudes towards animals between the public and those working with animals in livestock production [20,21,22]. There has not, however, been a validation of these tools for assessing animal-directed empathy in populations of people working directly with animals. The development and assessment of measures relevant to the target population can significantly alter the conceptualization of attitudes, including animal-directed empathy, and the associations between behaviours and decision making [23]. It is also known that humans’ animal-directed empathy can vary with different classifications of species [7]. Thus, there remains a need to enhance the evidence in support of validated measures of animal-directed empathy in diverse populations [16] and directly related to livestock.

The objectives of the current study were to enhance the evidence for using the AES and AES-SF in different populations and in relation to livestock species by (1) evaluating the validity and reliability of the AES and AES-SF in two populations (the general public and those involved in livestock production), and (2) developing and evaluating a short livestock empathy score (LES) relative to AES/AES-SF with the hypothesis that empathy as measured by species-specific questions would relate to the general latent concept of animal empathy.

## 2. Materials and Methods

The data collected for this study was approved by the University of Calgary research ethics board (REB21-1566, REB21-1809). Informed consent was obtained from all individuals involved in the study.

### 2.1. Participant Recruitment

Data was used from two studies evaluating perspectives and preferences of calf handling and restraint methods used during spring processing in western Canada. Spring processing is a period where calves are handled for procedures that support health and management, such as vaccinations, growth implants, ear tagging, castrations, and record keeping. Both studies used online surveys hosted on Qualtrics (Version 2022; Qualtrics, Provo, UT, USA). Study 1 involved an online survey of adults who participated in at least one beef calf processing event in the last 5 years and was conducted over a 4-month period [24]. Participants in Study 1 were recruited via a letter and QR code distributed by beef cattle and livestock associations, which resulted in 999 responses. Study 2 utilized a similar online survey; however, the population was a representative sample of 1006 members of the Canadian population stratified based on age and province of residence that were recruited and paid for their participation through the CloudResearch Prime Research Solutions [25].

### 2.2. Survey Tool

The 22 AES questions and additional 5 LES questions are presented in Table 1, with the scoring for each scale. Additional details of each survey can be found in [24,25]. Both surveys included a section with 27 five-point Likert-scale questions regarding empathy towards animals. The 27 questions consisted of the 22 questions from the original AES [17] and an additional 5 questions from studies specifically addressing empathy towards livestock or cattle for use in this analysis. These studies used language specific to cattle or farm animals and targeted empathy-related elements of empathetic concern [12], perspective taking [12], and connectedness [26,27].

### 2.3. Analysis

Data quality filters included straight-lining Likert scale questions for AES or LES (i.e., selecting the same response to all questions) and a general speed check. The general speed check was set at a threshold of a minimum of 5 min for the time required to complete the survey, including watching the videos, as described in [27]. Of the 999 responses in Study 1, 151 did not pass the speed check and 36 were removed due to straight-lining the AES/LES Likert scales, resulting in 812 survey responses for analysis. Study 2 had a total of 1006 responses, of which 338 were removed due to data quality issues (172 did not pass the speed check, 99 had more than one data quality issue, 28 were repeated individuals, 26 did not provide consent, and 13 straight-lined the AES/LES Likert questions), leaving 668 for analysis. Additional demographic information is available in [24,25].

The Animal Empathy Score was calculated according to the methods used by [17]. The additional five livestock-specific questions were used to create a livestock empathy score (LES). Responses to questions for the AES and LES were allocated higher scores for more empathetic responses, with questions representing empathetic sentiment having scores ranging from 5 to 9 for strongly disagreeing to strongly agreeing with the statements, and questions representing unempathetic sentiment being scored from 0 to 4 for strongly agreeing to strongly disagreeing. A final AES or LES score was created from the sum of the scores for individual questions. The scoring rubric is available in Table 1.

All data analysis was conducted using R [28]. To evaluate item selection for the scales, confirmatory factor analysis (CFA) was performed using a three-factor structure for populations from Study 1 and 2. While Likert items are ordinal variables, scales based on the sum of items can be treated as continuous variables for statistical analysis, particularly when the number of items is greater than 10 questions [29]. Prior to conducting the CFA, data was normalized within each population and assessed for suitability for factor analysis using the Kaiser–Meyer–Olkin measure of sampling adequacy [30] and Bartlett’s Test of Sphericity. Factors 1 and 2 were the same as those reported by [15] to create the AES-SF, and factor 3 consisted of the LES. Model fit was evaluated using the Comparative Fit Index (CFI) and the Root Mean Square Error of Approximation (RMSEA). McDonald’s omega was calculated as a measure of reliability. Convergent validity was evaluated by calculating the Spearman’s correlation coefficient between AES and AES-SF, and between the AES and LES scores separately for each population. Where the CFA did not fit the data, exploratory factor analysis was used.

## 3. Results

Detailed results of the sample populations are reported in [24,25].

### 3.1. AES and AES-SF—CFA, Reliability, and Convergent Validity

The KMO for the public population dataset was 0.92, indicating a marvelous adequacy of the dataset for factor analysis [30], and the Bartlett test for sphericity was also significantly different from the model correlation matrix (chi sq = 77,730.3, df = 435, *p* < 0.001), indicating that the data was suitable for factor analysis. The chi-squared test for the three-factor CFA in the public survey population indicates a good fit with the data (q = 3067.5, df = 78, *p* < 0.001). The CFI was 0.918, indicating a good fit of the three-structure model to the data. The RMSEA for this model was 0.07, which supports the consideration of the model for fitting the data [31]. All factor loadings and variances were significantly different from zero (Table 2). The McDonald omega values for all three factors were high.

In the population of participants in beef calf processing events, the KMO was 0.61, indicating a mediocre suitability of the dataset for factor analysis, and the Bartlett test for sphericity was also significant (chi sq = 87,745.8, df = 435, *p* < 0.001); however, the three-factor CFA model was insignificant using the chi-sq statistic for the model (*p* = 0.7). The exploratory factor analysis indicated a four-factor structure; however, all factor loadings were low (<0.5) and the cumulative proportion of variance explained was also very low (0.09).

The correlation between AES/AES-SF was high within the public population (rho = 0.92, *p* < 0.001) and moderately high within the industry population (rho = 0.62, *p* < 0.001).

### 3.2. LES—CFA, Reliability, and Convergent Validity

In the three-factor CFA, Factor 3 represented the LES. In the public population, this factor explained a significant amount of the variance and had a relatively high value for reliability, as measured by the McDonald omega statistic. The validity of LES in relation to general animal empathy, as measured by the correlation between AES and LES, was moderate for the public respondents (rho = 0.62, *p* < 0.001) and low for the participants in beef calf processing events (rho = 0.10, *p* = 0.002).

## 4. Discussion

The objectives of the current study were to enhance the evidence for using the AES and AES-SF in different populations and in relation to livestock species. The results of this study support the findings of [17] in the reliability and validity of the Animal Empathy Scale Short Form (AES-SF) in a sample from the Canadian public population. Corroboration of these results supports the AES-SF as a useful tool for shortening the assessment of animal empathy in quantitative assessments of animal-direct empathy.

The results of the current study, however, caution against the use of AES, AES-SF, and LES in populations of individuals directly involved in livestock production, specifically in beef production in Western Canada. In this population, the AES-SF performed marginally well in relation to correlation to AES. The CFA indicates that the AES-SF factors did not fit the data well, meaning that there might be more to the latent construct of expression of animal-directed empathy in this demographic. The correlations between measurements were also consistently lower in those that were directly involved in livestock production. While some interpret lower scores on AES as evidence of reduced empathy towards animals [22], this can prove challenging when comparing results between populations given the complexity of animal-directed empathy in those working directly with livestock species [23,32] and low indicators of validity and reliability seen in this study. Psychometric measurement models must be carefully selected and well-fitted to the study population, otherwise the results may be misleading [23]. Qualitative results from the population in this study reported the presence of empathy towards livestock in consideration of preferences for handling practices [24]. While not directly assessing empathy towards different animals, it is well shown in the scientific literature that there are differences in how the general public and those involved in animal production express their values in relation to animal welfare [21,33,34,35], despite many shared values underlying the frameworks common in discussing animal welfare [36,37].

In a review of decades of research of farmer’s views of animal welfare, empathy was a consistent construct in how those directly involved in livestock production view animals, with multiple studies citing themes of biological functioning and instrumental value [9]. It is possible that the AES’s focus on the emotional content of empathy did not capture the cognitive factors considered by those directly involved in livestock production or in relation to animal-directed empathy of livestock species. The review by [9] also reported that the strength of empathy’s relation to other constructs varied with method, with stronger relationships reported when using visual or qualitative measurement of animal-directed empathy over self-reported quantitative measurements. Reference [32] is an exemplary study showing that results of a quantitative assessment of attitudes towards animals did not reflect that farmers viewed animal-directed empathy and instrumental value as connected in many ways. While the significant heterogeneity in methods influenced research outcomes, the issue of poor reliability and validity in the livestock production population in the current study supports the inference that self-reporting quantitative measurements validated in non-target populations are not suitable measures of animal-directed empathy as expressed by individuals directly involved in livestock production.

Attempts to measure animal-directed empathy towards different species or with those directly involved in livestock production often utilize the re-wording of questions to create statements with the subject being the farm animal of interest. The LES was an attempt to utilize a similar approach with fewer items, with the opportunity to evaluate construct validity relative to AES and in different populations. While construct validity was moderate in the public population, it was very low in the producer population. Ref. [18] also found low internal reliability with the use of the modified AES for dairy cattle when used with a population of people who work directly with dairy cattle. Attitude towards animals, which often has an empathic component, varies in public populations in relation to species and intended human use [38], and empathy towards animals has been reported to decrease with increasing time since a species’ evolutionary divergence from humans [7]. The questions used to formulate the LES, while from existing tools, together may not have captured the latent construct of empathy towards livestock species, particularly for individuals who work directly with these animals, or the population surveyed in this study may differ due to characteristics beyond their association with livestock production. The LES from this study may be useful for evaluating livestock specific empathy in public populations but is likely not appropriate for populations involved in livestock production.

This study utilized results from two online surveys of the target populations. The limitations of self-reported quantitative assessment restricted the ability to evaluate further details on the variation in the construct of animal-directed empathy associated with species and target population. The tools used in this and many studies were developed using populations not involved in livestock production, and thus the language used may not represent the vernacular of those involved with livestock production. Previous work showed that empathy towards animals was a core theme in the evaluation of handling methods for calves in participants of this study [24]. Therefore, the poor model fit may be due to the language of the tool being misaligned with language used by this population to express empathy towards animals. In both the public and participant populations, further work is needed to evaluate whether similar or divergent language is used to express empathy towards livestock species and if alternative language would result in a better model fit. Future studies should utilize qualitative interviews to determine whether revising the language of empathy measurement tools would improve the fit of these tools for the species of interest and different populations. Convergent validation with other constructs of attitudes towards animals and personality traits associated with empathy was not possible in the current data collection due to survey length restrictions and a focus on different topics. Similarly, a comparison with other self-reported quantitative measures, such as the ETA, would provide researchers with more robust criteria for the selection of animal-directed empathy measures. The current validity and reliability assessment reflects a single point in time in both populations, which is limited due to an inability to evaluate test/re-test reliability and consistency over time. There may also be differences due to the geographic region of the survey for participants in beef production and the species of focus in the survey (beef calves), which led to differences in the suitability of the data for the models.

## 5. Conclusions

The focused development of animal-directed empathy measurements must be considerate of the species of subject, as well as the population being assessed. The results of the current study indicate that the use of AES or AES-SF in public populations is reliable and valid, confirming the results of [15]. However, while the AES-SF correlated well with the AES in the population directly involved in livestock production, the structure of the AES-SF did not fit well according to CFA and therefore should be used with caution, if at all, in these populations.

The LES tool from this study may be useful in measuring empathy towards livestock in public populations, but more work is needed to develop a measurement relevant in populations involved in livestock production. The validity and reliability of measurements in the population directly involved in livestock production supports the inference that self-reported quantitative measurements validated in non-target populations are not suitable measures of animal-directed empathy as expressed by individuals directly involved in livestock production.

## Figures and Tables

**Table 1:** Scoring rubric for Animal Empathy Score and Livestock Empathy Score questions ^1^.

Question Number	Question Language	Empathetic Sentiment	Response Scoring ^2^					Species
			Strongly Agree	Agree	Neutral	Disagree	Disagree Strongly	
1	So long as they’re warm and well fed, I don’t think zoo animals mind being kept in cages.	Negative	0	1	2	3	4	Wildlife
2	Often cats will meow and pester for food even when they are not really hungry.	Negative	0	1	2	3	4	Pets
3	It upsets me to see animals being chased and killed by lions in wildlife programs on TV.	Positive	9	8	7	6	5	Wildlife
4	I get annoyed by dogs that howl and bark when they are left alone.	Negative	0	1	2	3	4	Pets
5	Sad films about animals often leave me with a lump in my throat.	Positive	9	8	7	6	5	Animal
6	Animals deserve to be told off when they’re not behaving properly.	Negative	0	1	2	3	4	Animal
7	It makes me sad to see an animal on its own in a cage.	Positive	9	8	7	6		Animal
8	People who cuddle and kiss their pets in public annoy me.	Negative	0	1	2	3	4	Pets
9	A friendly purring cat almost always cheers me up.	Positive	9	8	7	6	5	Pets
10	It upsets me when I see helpless old animals.	Positive	9	8	7	6	5	Animal
11	Dogs sometimes whine and whimper for no real reason.	Negative	0	1	2	3	4	Pets
12	Many people are over-affectionate towards their pets.	Negative	0	1	2	3	4	Pets
13	I get very angry when I see animals being ill treated.	Positive	9	8	7	6	5	Animal
14	It is silly to become too attached to one’s pets.	Negative	0	1	2	3	4	Pets
15	Pets have a great influence on my moods.	Positive	9	8	7	6	5	Pets
16	Sometimes I am amazed how upset people get when an old pet dies.	Negative	0	1	2	3	4	Pets
17	I enjoy feeding scraps of food to the birds.	Positive	9	8	7	6	5	Wildlife
18	Seeing animals in pain upsets me.	Positive	9	8	7	6	5	Animal
19	People often make too much of the feelings and sensitivities of animals.	Negative	0	1	2	3	4	Animal
20	I find it irritating when dogs try to greet me by jumping up and licking me.	Negative	0	1	2	3	4	Pets
21	I would always try to help if I saw a dog or puppy that seemed to be lost.	Positive	9	8	7	6	5	Pets
22	I hate to see birds in cages where there is no room for them to fly about.	Positive	9	8	7	6	5	Pets
23	I feel sorry for cattle when they have problems or suffer.	Positive	9	8	7	6	5	Livestock
24	I sometimes try to understand farm animals better by imagining how things look from their perspective.	Positive	9	8	7	6	5	Livestock
25	I am curious about cattle.	Positive	9	8	7	6	5	Livestock
26	If I see someone hurt a farm animal I feel compassion for it.	Positive	9	8	7	6	5	Livestock
27	Cattle’s misfortunes do not disturb me a great deal	Negative	0	1	2	3	4	Livestock

^1^ Questions 1–22 are the original AES from [14]. Questions 7, 8, 10, 13, 14, 16, 18, and 19 were used for the AES-SF as created by [15]. Questions 23–27 were questions taken from [12,26,27] to create the Livestock Empathy Score. ^2^ Response scoring is the value assigned to each Likert scale response for each question. The value corresponding to an individual’s response was assigned for each question, and then summed for all questions assigned to the AES, AES-SF, and LES to determine an individual’s score.

**Table 2:** Results from a three-factor confirmatory factor analysis in a sample from members of the Canadian general public.

AES Question	Factor 1 Loading ^1^	Factor 2 Loading ^1^	Factor 3 Loading ^2^	Item–Total Score Correlation
7	1			0.54
10	1.43			0.67
13	1.21			0.60
18	1.51			0.70
8		1		0.58
14		1.18		0.66
16		0.99		0.56
19		1.15		0.68
23			1	0.67
24			0.71	0.50
25			0.58	0.42
26			1.01	0.69
27			0.95	0.69
Cumulative Proportion of Variance Explained	0.26	0.46	0.5	
McDonald’s Omega	0.75	0.82	0.75	

^1^ Factors 1 and 2 represent factors in the AES-SF from [15]. ^2^ Factor 3 consists of questions from the LES.

## Data Availability

The datasets presented in this article are not readily available because of participant confidentiality.
